# Supplementary material for: Immunohistochemical analysis of CD155 expression in triple-negative breast cancer patients
Source: PLoS One. 2021 Jun 11;16(6):e0253176. doi: 10.1371/journal.pone.0253176 (PMC8195407; doi:10.1371/journal.pone.0253176)
Supplement: S1 Table — (PDF) [file pone.0253176.s001.pdf]

No.	Age	menoposal status	pathological maximum invasive compornent (mm)	positive lymph nodes	pTNM	pStage	histological grading	ly	v	Ki-67(Ope)	Adjuvant chemotherapy	CD155	Stromal TILs (LPBC)	PD-L1 on immune cells(73-10)	PD-L1 on immune cells (SP142)	PD-L1 on tumor(SP142)	PD-L1 on tumor(73-10)	RFS(month)	relapse	OS(month)	death
5	75	-	15	0	T1cN0M0	I	3	+	-	40	+	-	-	+	+	-	-	20		20	
7	31	+	12	0	T1cN0M0	I	3	-	-	70	+	+	+	+	+	+	+	20		20	
15	41	+	20	0	T1cN0M0	I	3	-	+	40	+	-	-	+	+	-	-	35		35	
16	68	-	18	0	T1cN0M0	I	3	-	-	70	+	+	+	+	+	+	+	36		36	
21	66	-	22	0	T2N0M0	II A	2	+	-	20	+	-	-	-	-	+	-	31		31	
42	83	-	18	2	T1cN1M0	II A	3	-	-	70	-	+	-	+	-	+	-	18		18	
44	76	-	18	0	T1cN0M0	I	2	-	-	40	-	+	+	+	-	-	+	40		40	
57	77	-	6	0	T1bN0M0	I	1	-	-	15	+	-	-	-	-	-	-	34		34	+
59	65	-	10	2	T1bN1M0	II A	2	+	-	25	+	+	+	+	+	+	+	53		53	
62	77	-	5	0	T3N0M0	II B	3	+	+	80	-	+	-	+	-	+	-	11	+	23	
63	68	-	20	0	T1cN0M0	I	2	+	-	45	-	+	-	+	-	-	+	71		71	
65	62	-	28	0	T2N0M0	II A	2	+	-	38	+	+	+	+	+	+	+	70		70	
66	74	-	35	9	T2N2M0	III A	2	+	+	50	-	-	+	+	+	+	+	18	+	36	+
67	54	-	22	0	T2N0M0	II A	2	+	+	15	+	+	-	-	-	-	-	69		69	
70	80	-	12	0	T1bN0M0	I	2	+	+	20	-	-	-	+	-	-	-	61		61	
72	93	-	25	2	T2N1M0	II A	2	+	+	40	-	+	-	+	-	-	+	31		31	
73	60	-	42	0	T2N0M0	II A	2	+	+	80	+	+	+	+	+	+	+	66		66	
76	87	-	55	not tested	T4a cN0M0	III B	3	+	+	40	-	+	-	-	-	-	+	22		29	+
79	73	-	38	0	T2N0M0	II A	3	+	+	55	undetermined	+	-	+	+	-	-	44		44	
81	84	-	40	0	T2N0M0	II A	3	+	+	80	-	+	-	+	-	-	-	57		57	+
85	45	unknown	20	0	T1cN0M0	I	3	+	+	70	+	-	-	+	+	-	+	11		11	
86	67	-	20	0	T1cN0M0	I	3	+	+	50	+	-	-	+	-	-	+	95		95	
87	71	-	16	1	T1cN1M0	II A	2	+	-	70	-	+	-	+	+	-	-	9	+	18	+
88	70	-	12	0	T1cN0M0	I	2	+	-	28	-	+	+	+	+	+	-	91		91	
89	60	-	13	not tested	T1c cN0M0	I	2	+	-	80	-	-	-	+	+	-	-	76		76	
90	88	-	40	2	T2N1M0	II B	2	+	+	50	-	-	-	-	-	-	-	22	+	26	+
91	58	-	20	not tested	T1b cN0M0	I	3	+	+	50	+	-	+	-	+	+	-	111		111	
93	44	+	27	3	T2N1M0	II B	3	+	+	47	+	+	-	+	-	-	-	50		50	
94	69	-	54	0	T4bN0M0	III B	2	+	-	40	undetermined	-	-	-	-	-	-	73		73	
97	70	-	10	0	T1bN0M0	I	1	+	-	25	+	-	-	+	-	-	-	107		107	
99	37	+	50	2	T3N1M0	III A	3	+	+	80	+	-	-	-	-	-	-	9	+	16	+
100	80	-	21	0	T2N0M0	II A	3	+	+	80	+	-	-	-	-	-	-	68		68	
102	61	-	40	5	T2N2M0	III A	3	+	+	25	+	-	-	-	-	-	-	48	+	80	+
103	37	+	30	0	T2N0M0	II A	3	+	+	40	+	-	-	-	-	-	+	52		52	
110	74	-	25	0	T2N0M0	II A	2	+	+	40	+	-	+	+	+	-	+	97		97	
111	78	-	40	3	T2N1M0	II B	3	+	+	49	+	-	-	-	-	-	-	61		61	
118	89	-	35	not tested	T2 cN0M0	II B	3	+	+	5	-	-	-	-	-	-	-	46		46	
119	67	-	7	not tested	T1b cN0M0	I	2	+	-	60	+	-	-	+	+	+	+	64		64	
125	60	-	6	not tested	T1b cN0M0	I	2	-	-	not tested	-	-	-	-	not tested	not tested	-	80		80	
127	55	-	50	0	T2N0M0	II A	3	+	+	15	undetermined	-	-	-	+	-	+	71		71	
129	44	+	30	not tested	T2 cN0M0	II A	3	+	-	30	+	+	+	+	+	+	+	72		72	
131	36	+	13	0	T1cN0M0	I	2	+	-	70	+	+	-	+	+	-	-	126		126	
138	62	-	26	0	T2N0M0	II A	2	+	+	10	+	-	+	+	-	-	-	143		143	
140	67	-	25	0	T2N0M0	II A	2	+	+	10	+	-	-	+	+	-	-	142		142	
141	81	-	18	2	T1cN1M0	II A	2	+	+	15	+	-	-	-	-	-	-	111		111	+
143	82	-	12	not tested	T1c cN0M0	I	3	+	+	30	-	-	-	-	-	-	-	31		31	
144	74	-	23	not tested	T2 cN0M0	II A	2	+	-	70	-	-	-	+	-	-	-	99		99	
145	62	-	2	not tested	T1a cN0M0	I	2	-	-	not tested	-	-	+	-	-	-	-	94		94	
147	80	-	40	1	T2N1M0	III B	3	+	+	30	-	+	-	+	+	+	+	56		56	+
148	64	-	35	4	T2N2M0	III A	2	+	+	15	+	-	-	-	-	-	-	40	+	72	
149	69	-	25	0	T2N0M0	II A	3	+	+	25	+	-	+	+	+	+	+	155		155	
151	79	-	22	not tested	T2 cN0M2	II A	3	+	+	50	-	-	-	-	-	-	-	60		60	+
152	54	-	18	0	T1cN0M0	I	3	+	+	60	+	+	-	-	-	-	-	131		131	
153	58	-	20	not tested	T1b cN0M0	I	3	+	+	15	+	-	-	-	-	-	+	35	+	42	+
156	50	-	20	18	T1cN3M0	III C	3	+	+	70	-	-	+	+	+	-	+	11	+	41	+
157	41	+	17	not tested	T1b cN0M0	I	3	+	+	60	+	+	+	-	-	-	-	150		150	
159	58	-	2	0	T1aN0M0	I	3	+	-	not tested	+	-	+	-	-	-	-	48		48	
161	52	-	20	0	T1cN0M0	I	3	+	+	80	+	+	+	+	+	-	+	101		101	
162	45	+	25	0	T2N0M0	II A	3	+	+	not tested	-	+	-	+	-	-	-	15	+	57	+
163	71	-	12	not tested	T1b cN0M0	I	2	+	-	60	-	+	-	+	-	-	+	53		53	
168	81	-	40	0	T2N0M0	II A	2	-	-	15	-	-	+	+	+	-	+	16		16	
